# Gene Fusion Markup Language: a prototype for exchanging gene fusion data

**DOI:** 10.1186/1471-2105-13-269

**Published:** 2012-10-16

**Authors:** Shanker Kalyana-Sundaram, Achiraman Shanmugam, Arul M Chinnaiyan

**Affiliations:** 1 Michigan Center for Translational Pathology, University of Michigan Medical School, Ann Arbor, MI, 48109, USA; 2 Department of Environmental Biotechnology, Bharathidasan University, Tiruchirappalli, India; 3 Department of Pathology, University of Michigan Medical School, Ann Arbor, MI, 48109, USA; 4 Howard Hughes Medical Institute, University of Michigan Medical School, Ann Arbor, MI, 48109, USA; 5 Department of Urology, University of Michigan Medical School, Ann Arbor, MI, 48109, USA

## Abstract

**Background:**

An avalanche of next generation sequencing (NGS) studies has generated an unprecedented amount of genomic structural variation data. These studies have also identified many novel gene fusion candidates with more detailed resolution than previously achieved. However, in the excitement and necessity of publishing the observations from this recently developed cutting-edge technology, no community standardization approach has arisen to organize and represent the data with the essential attributes in an interchangeable manner. As transcriptome studies have been widely used for gene fusion discoveries, the current non-standard mode of data representation could potentially impede data accessibility, critical analyses, and further discoveries in the near future.

**Results:**

Here we propose a prototype, Gene Fusion Markup Language (GFML) as an initiative to provide a standard format for organizing and representing the significant features of gene fusion data. GFML will offer the advantage of representing the data in a machine-readable format to enable data exchange, automated analysis interpretation, and independent verification. As this database-independent exchange initiative evolves it will further facilitate the formation of related databases, repositories, and analysis tools. The GFML prototype is made available at http://code.google.com/p/gfml-prototype/.

**Conclusion:**

The Gene Fusion Markup Language (GFML) presented here could facilitate the development of a standard format for organizing, integrating and representing the significant features of gene fusion data in an inter-operable and query-able fashion that will enable biologically intuitive access to gene fusion findings and expedite functional characterization. A similar model is envisaged for other NGS data analyses.

## Background

Gene fusions are well-recognized molecular events and serve as genetic markers and drug targets for several hematological disorders [1,2]. The discovery of recurrent ETS-family translocations in prostate cancer [3,4], a RAF kinase gene fusion in ETS-negative prostate cancer [5,6], and an ALK kinase fusion in lung cancer [7] further advocates the significance of gene fusion events in the development of epithelial cancers [8,9]. The discovery of gene fusion candidates was infrequent and challenging because of various technological limitations inherent in traditional techniques, such as spectral karyotyping, comparative genomic hybridization (CGH), representational oligonucleotide microarray analysis (ROMA), fluorescent *in situ* hybridization (FISH), and Sanger-based sequencing [10]. However, the rapid evolution of non-Sanger-based massively parallel sequencing technologies has empowered an unprecedented sequencing speed, enabled an unbiased systematic characterization of large-scale genome-wide analysis, and surprised researchers with numerous gene fusion candidates at an unmatched resolution [11-13]. As this development in sequencing technology is still relatively new, no standard approach has been established for reporting or documenting a number of the key features associated with gene fusion discovery leading to a repetitive process of manual curation and interpretation of critical information that can entail scrutinizing the entire manuscript along with the supplementary information. To enable researchers to remain current with the information flow and make the data more accessible, various non-standard community efforts have been established through other structural variation databases [14-17]. These were primarily developed as independent entities without much scope for data exchange and integrity. Moreover, the non-standard mode of reporting the rapidly increasing gene fusion discoveries has challenged their manual processes of curation and data entry when updating the database. Finally, a number of existing databases are not suited for documenting gene fusion features and lack many of the key attributes that are required for next-generation sequencing (NGS) analyses. In the near future, as we anticipate a downpour of gene fusion candidates from various high throughput analyses, representing the data in an unstructured, autonomous format poses a significant risk of misplacing such valuable information. Similar to other standardization efforts and procedures such as the Minimum Information About a Microarray Experiment (MIAME) that standardizes and shares microarray data [18], the Proteomic Standard Initiative-Molecular Interaction (PSI-MI) that was established to regulate protein-protein interaction representation [19], and the Systems Biology Markup Language (SBML) that represents biochemical reaction networks [20], there is an urgent need to create a standard for recording and reporting gene fusion discoveries. Here, we propose a prototype standardization initiative we call the Gene Fusion Markup Language (GFML) to propose a standard format for representing and documenting gene fusions. In turn, this improved documentation will enable rapid access and maximize the usage of gene fusion findings to other researchers in the community. Hence, these proposed guidelines would serve as a starting point for stricter standards that will evolve over time in the field as the standards undergo regular and thorough vetting by the community [21].

### Current literature issues

**Figure 1:**
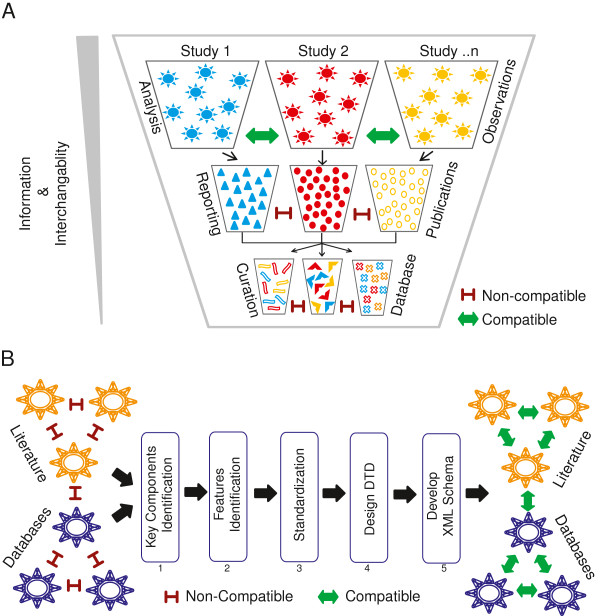
** A) Schematic representation of information and interoperability loss across various stages of communication (from top to bottom).** In the first tier, the trapezoids or cones represent different studies, while the complex shapes (circles + triangles) shown in various colors represent the parallel observations derived at the analyses level across each study. The input and output at each tier is indicated at the left and right respectively. The individual shapes (triangles and circles) in the second tier denote the partial and heterogeneous representation of information carried over to the publication level from the respective analyses of each study. Further changes taking place is reflected in the shapes in the third tier and represent the reduced and incompatible data across each study curated from the literature. Information and interoperability loss is indicated by the grey triangle on the left. The double-headed green arrows and red blocks indicate the compatibility status. **B**) Schematic representation of the steps (from left to right) in the GFML workflow. 1 & 2: Identification of key components and features of NGS gene fusion analyses from literature evidence and database curation not readily compatible, 3: Element and attribute standardization, 4 & 5: Implementation of XML Schema. Double-headed green arrows and red blocks indicate the compatibility status.

Researchers have tried to practice and adopt the best possible methods to present their gene fusion data to the community. However, no standard list of features or data structures has been discussed or adopted to represent this special category of observations in an inter-operable and query-able fashion. In most cases, authors tend to describe the gene fusion and its features based on a fusion detection algorithm used for a particular set of studies and to represent the information based on their own experience. The depth of information generated from an NGS analyses pipeline, mostly filtered out in the published literature, leads to significant information loss between the observed and reported findings (Figure 1A). Here, we summarize some of the key concerns. 1. Representation Format: In published reports data is simplified and filtered to include only the disease-specific or study-related gene fusions are described in an informal format (e.g., a colorful schematic representation). Other non-recurrent or biologically unrelated gene fusions for the given study are merely listed in the Results or the Discussion of the article as a running text or are at times represented pictorially as a Circos plot, histogram, or table (as an image). Besides explaining the significance of the observations, there is no additional effort to represent the complete analyses outcome in a consistent and sharable manner. 2. Splice coordinates: Apart from the fusion genes considered significant for the respective study, most of the other gene fusion candidates are simply listed in a table along with the number of supporting reads. Unfortunately, the features that help predict their significance are not described. In order to assess the true biological significance of candidate fusion transcripts, the splice coordinates (exon junction) are essential for the interpretation of the reading frame and domain signature of the fusion product. For example, the significance of fusion candidates involving kinase family members or transcription factors can be easily misinterpreted if there is no functional domain retained in the open reading frame of the fusion transcript. 3. Read evidence: To the best of our knowledge, there is no published study that reports actual reads mapped to any candidate gene fusion. Currently, the only way to access the gene fusion-specific reads is to download the large raw sequence files from public sequence repositories (only if available and accessible) and perform the non-trivial task of repeating the complete analyses that can entail everything from mapping the raw reads to a fusion discovery pipeline. As there are many fusion candidates predicted in each study with potential false-positives, it is vital to retain sequence evidence from the analysis phase so that the observations can be independently verified. 4. Terminology: A controlled vocabulary in presenting the characteristic features of gene fusion events and their evidence is another potential problem that needs to be addressed in this discipline. Almost half a dozen published fusion detection algorithms have been independently developed. These algorithms are associated with a number of discrepancies in naming the attributes and values describing gene fusion events and the supporting sequence evidence [22-28]. 

### Existing database issues

There are some common evolving standards towards representation of generic structural variations [29], but there is no specialized data exchange standard available to submit published gene fusion discoveries with required NGS features to any public repositories or databases. In order to avoid repetitive manual curation and enhance data accessibility, there have been independent efforts to curate and document these gene fusion discoveries in public databases. The Database of Genomic Variants [17,29], the Mitelman Database of Chromosome Aberrations and Gene Fusions in Cancer [16], the Atlas of Genetics and Cytogenetics in Oncology and Hematology [15], and the Catalogue of Somatic Mutation in Cancer [14] are a few of the most popular and closely related databases of this kind. Here, we summarize some of the key concerns with the existing resources. 1. NGS incompatible: NGS features that are specific to gene fusions are ignored and not captured, with the current gene fusion entries forcefully accommodated under generic structural variations. In other words, most of the existing databases are not yet adapted for NGS analyses. For example, information pertaining to the sequencing platform, mapping, the fusion detection algorithm, and the read evidence are omitted, leading to yet another level of information loss when the information from the published literature is captured in databases (Figure 1A). 2. Non-interoperable: Most of the existing online databases function independently with different scopes of operations and report curated data in custom formats not compatible with one another. In turn, this interrupts the data exchange and integrity (Figure 1A). 3. Unsynchronized update: Existing databases provides some function to the community. However, considering the volume of information generated out of recent NGS analyses and the current status of non-standard data representation in the literature, the manual mode of operation involved in curating and documenting such massive amount of data may significantly hamper the up-to-date status of the database. 4. Incomplete entries: Manual curators primarily focus on validated gene fusions therefore many other non-validated candidates listed in the supplementary materials are overlooked and are represented in only a few of the current databases. Moreover, the majority of gene fusion candidates predicted from NGS studies are listed in the supplementary materials because of the limited space allowed by many journals.

### Requirement for specialized standard data exchange format for gene fusions

Various standardization procedures have been adopted by the community periodically, each designed to handle various data types generated by different technologies that is appropriate for the level of information required for the exchange. For example, BioXSD is a common generic data exchange format that bridges the gap between specialized standardization formats [30]; specialized data formats such as MAGE-ML are useful in describing the minimum information specific to microarray experiment [18]; PSI-MI is more applicable for handling information specific to protein-protein interactions [19]; and Molecular Methods (MolMeth) database provides the research community with an up-to-date source of methods and protocols used in molecular biology and medicine [31]. Similarly, several sequencing-based standardization formats have been developed by open-access and international working bodies such as Genomic Standards Consortium (MIGS\MIMS\MIENS\MIMARKS\MIxS) and European Nucleotide Archive (ENA\SRA) that describe genomic, meta-genomic, and environmental sequences [32-36]. Previously developed sequencing-based standardization methods primarily focused on basic data such as sample information, experimental setup, machine configuration, sequence traces, reads, quality scores, assembly, mapping and annotation. However, standardization procedures describing secondary/tertiary analyses and their outcomes are limited, especially in the context of rapidly evolving technological advances in next generation sequencing. The currently available Minimum Information about a high-throughput Nucleotide SeQuencing Experiment (MINSEQE), extends the MIAME specifications to capture the quantitative data (expression) from HTS technology, while dbVAR and GVF are specialized data formats to exchange genomic structural variations [37,38]. Similarly, we envision a customized standardization procedure to accommodate the features of gene fusion data arising from the transcriptomic analyses that incorporates valuable attributes such as description of fusion detection algorithms, 5’ and 3’ fusion transcript annotations, fusion read evidence, open reading frames, splice junction features, experimental validation status, functional domain architecture, etc. currently not provided in a common data exchange format. Many of the common attributes of gene fusion schema could potentially be derived from other XML Schemas (SRA\ENA, PSI-MI, MAGE-ML), such as “Study”, “Sample”, and “Experiment” but the descriptions of such elements are also often varied across existing standardizations and are primarily based on the platform\ technology (sequencing, Co-IP, array-based, etc.,) that are specifically suited for the generation of information of primary interest. For example, the element “Experiment” is fine-tuned to fit sequencing platform details in SRA to exchange sequence data, in PSI-MI it is structured to handle protocols which generate protein-protein interactions, and in MAGE-ML it is designed to capture information from array based technologies. Therefore in the proposed GFML prototype, in order to ensure a wide scope for curation of gene fusion features across diverse platforms including NGS, FISH, aCGH, qPCR, etc., the common elements have not been derived from any of the available individual standards. However, to take advantage of the existing schema, tools to facilitate cross-talk and data sharing between various schemas will be enabled.

## Methods

We initiated an investigation to understand the commonality and characteristics of the published features and format in NGS studies specifically involving gene fusion algorithms and related findings [5,6,22-28,39,40]. We identified nine major elements and related attributes describing the characteristics of gene fusion events and the sequence evidence. We also investigated the existing structural variations databases [14-17,39] to understand the documented features, working model, and current status of each database. To design and develop a compatible and robust data model, we also adopted some of the relevant features of existing standardization protocols and markup languages [19]. Based on our interpretation of existing sources, we standardized the identified elements and features and further developed a prototype (Additional files 1 and 2). The graphical version of the GFML prototype was made using Altova XMLSpy version 2011rel3sp1 ( http://www.altova.com/). The complete process flow is represented in Figure 1B.

## Results

**Figure 2:**
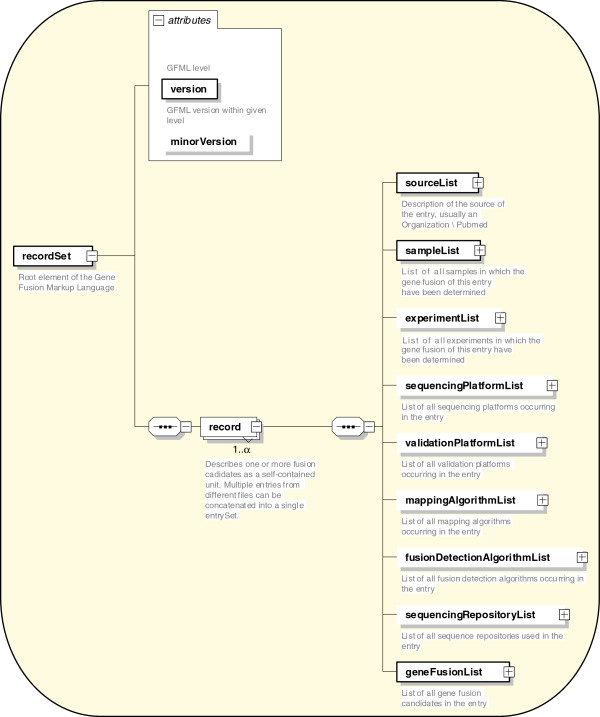
** Graphical representation of the Gene Fusion Markup Language structure.** Nine major elements are represented in the figure; the minor elements and their features can be viewed from the GFML. Mandatory elements are denoted by boxes with solid outlines and optional elements with open boxes. The plus sign indicates that the parent elements have child elements that are not shown; “1…infinite” denotes the one or many child elements that can occur.

In addition to specifying all required data elements and features, the model recommends a database-independent structure and standardizes the data attributes and its values. The ultimate goal of this model is to provide a standard framework that enables different types of complex, biologically meaningful queries in order to maximize the data usage of the system (Table 1). “Record_Set” is the root element of the GFML that contains one or more records (Figure 2). Each “Record” is a self-contained unit that allows gene fusion entries from one to multiple sources. Nine major elements have been characterized and identified from various transcriptome studies describing gene fusion. A brief introduction for each element is summarized as follows (detailed technical comments are available in the XML documentation): (1) the “Source_List” element allows one or more source objects, describes the source of the study and normally refers to the publication or data provider details; (2) the “Sample_List” allows one or more sample objects and describes the properties of the biological specimens associated with the study; (3) the “Experiment_List” element allows one or more experiments and describes the experimental parameters associated with the study; (4) the “Sequence_Platform_List” element allows one or more sequence platform objects and describes the required sequencing parameters such as the platform name, description and application type; (5) the “Validation_Platform_List” elements allow one or more validation platform objects and describe the validation methods; (6) the “Mapping_Algorithm_List” allows one or more mapping algorithm objects and describes the mapping algorithm and its parameters; (7) the “Fusion_Detection_Algorithm_List” allows one or more fusion detection algorithm objects and describes the fusion algorithm and its parameters; (8) the “Sequence_Repository_List” elements allow one or more sequence repository objects and describe the sequence repository details where the raw sequences have been deposited and made accessible; and (9) the “Gene_Fusion_List” element allows one or more gene fusion objects that describe the key attributes pertaining to gene fusion analyses including chromosomal location, gene annotation, read evidence, ORF status, the splice junction, potential mechanism, and validation status and is linked to other elements in the model, including the experiment, sample, mapping, and the fusion detection algorithm. For accommodating the available legacy data, the initial version of the prototype has been made highly flexible. Only 3 out of 9 elements (i.e., “source,” “sample,” “gene fusion”), represented as a thick line in Figure 2, are considered mandatory, whereas all others are represented as optional elements. As a proof of concept, we further illustrated the usefulness of this model with a prostate cancer-specific gene fusion candidate *TMPRSS2-ERG,* re-discovered by next generation sequence analyses [26] (Additional files 3 and 4). The instance further validated using the defined XML Schema Definition (Additional files 1 and 2). 

**Table 1:** Potential biologically relevant queries enabled by GFML

	**Usage scenario**	**Example**
**Clinicians**	Gene identifier	Lists fusion candidates that involve ERG
	Sample type	Lists prostate tissue candidates
	Clinical significance	Lists leiomyosarcoma candidates
	Reference	Pubmed ID/author information
**Molecular Biologists**	Protein domain	Lists fusion candidates with kinase domain
	Break in specific domain	Lists fusion that breaks or loses the SH2\SH3 domain
	Domain with specific region	Lists fusion candidates with 3’ kinase domain
	ORF status	Lists fusion candidates with potential ORF
	Validation status platform	Lists fusion candidates with validation status by FISH
	Splice pattern	Lists non-canonical splice pattern
	Fusion product	Lists potential fusion proteins/truncated proteins
	Fusion mechanism	Lists potential inversion
	Splice boundary	Lists 5’ inter-genic fusion (splice boundary)
**Bionform-aticians**	Sequencing platform	Lists candidates from Illumina
	Sequence evidence	Lists candidates with sequence evidence
	Mapping/fusion algorithm	Lists candidates detected using Bowtie + Shortfuse algorithm
	RPKM/unique count	Lists candidates above 50 RPKM

### Controlled vocabularies

A central requirement for efficient data exchange is a common data exchange format, however the presence of this format is not a guarantee of data compatibility. Ensuring the standardized use of the data attributes and its values through documentation and controlled vocabularies is also essential. Akin to other standardization protocols, controlled vocabularies related to gene fusion attributes and attribute values are subjected to initial standardization in order to enhance the dynamic queries. To avoid redundancy the common elements such as sample details, experimental design, and protocol description that have already been discussed and standardized by other efforts were avoided [18,19,32,33,36,40] and emphasis is placed only on the new data elements specific to next generation sequencing and gene fusion analyses. We believe there are much room to improve upon these vocabularies as the system progresses and becomes widely adopted by the community.

## Discussion and conclusions

Each successful surveying information system that developed and supported by a community has adopted, during its evolution, certain standard procedures that were warranted for data integrity and interoperability. Although a vast amount of microarray data and protein-protein interaction data were generated and reported in the public domain in a non-standard manner, over time the impaired status of data access and integrated analyses were exposed. In response to these issues, communities designed and developed appropriate standardization procedures such as MIAME / MAGE-ML [18] and PSI [19]. A number of gene fusion candidates from NGS studies have been recently reported without conforming to any standardized procedures for describing and documenting the associated data. Because of this lack of standardization, there is an enormous risk of inaccessibility of such valuable information that can consequently impede data integrity and downstream analyses. Although the NGS studies are new and still evolving, the rapid generation of information underscores the immediate requirement for a standardization procedure to represent and document the data in a common format for publication in a journal and deposition in a database in an exchangeable fashion. We believe that our proposed prototype standardization tool, Gene Fusion Markup Language (GFML) helps in resolving existing inconsistencies in gene fusion data representation and will facilitate the development of interoperable data model that can be dynamically queried and interpreted across different systems.

### Future plans

The model presented here offers the first necessary steps towards standardization of gene fusion features to share and exchange in a common standard format among the research community. The concept of incorporating the secondary and tertiary features derived from high throughput data can be extended to NGS-based *de novo* sequencing, gene expression, epigenetics, copy number variation, comparative genomics, metagenomics and pathogens. Collaboration with other standardization consortia will be pursued to further develop and extend the existing standards. As part of the community standard initiative, we intend to engage the research community in discussions and look forward to active participation by others to catalyze future development.

## Competing interests

The authors declare that they have no competing interests.

## Authors’ contributions

SK-S, AS and AMC designed the study and prepared the manuscript. All authors read and approved the final manuscript.

## Supplementary Material

Additional file 1 GFML_Schema.XSD – Describes the XML Schema Definition (XSD) of Gene Fusion Markup Language, can be viewed in internal explorer or in any XML editor. Click here for file

Additional file 2 GFML_common.XSD – Describes the common reusable elements of Gene Fusion Markup Language, can be viewed in internal explorer or in any XML editor. Click here for file

Additional file 3 GFML_prototype_instance.XML – Demonstrate the Gene Fusion Markup Language prototype with illustration, can be viewed in internal explorer or in any XML editor. Click here for file

Additional file 4 ** GFML_prototype.XML – XML document describes the prototype of the Gene Fusion Markup Language.** It would be good to view in internal explorer or in any XML editor. Click here for file

## References

[B1] Nowell PC The minute chromosome (Phl) in chronic granulocytic leukemia Blut 1962 8 65 66 14480647 10.1007/BF01630378

[B2] de Klein A van Kessel AG Grosveld G Bartram CR Hagemeijer A Bootsma D Spurr NK Heisterkamp N Groffen J Stephenson JR A cellular oncogene is translocated to the Philadelphia chromosome in chronic myelocytic leukaemia Nature 1982 300 5894 765 767 6960256 10.1038/300765a0

[B3] Tomlins SA Laxman B Dhanasekaran SM Helgeson BE Cao X Morris DS Menon A Jing X Cao Q Han B Distinct classes of chromosomal rearrangements create oncogenic ETS gene fusions in prostate cancer Nature 2007 448 7153 595 599 17671502 10.1038/nature06024

[B4] Tomlins SA Rhodes DR Perner S Dhanasekaran SM Mehra R Sun XW Varambally S Cao X Tchinda J Kuefer R Recurrent fusion of TMPRSS2 and ETS transcription factor genes in prostate cancer Science 2005 310 5748 644 648 16254181 10.1126/science.1117679

[B5] Palanisamy N Ateeq B Kalyana-Sundaram S Pflueger D Ramnarayanan K Shankar S Han B Cao Q Cao X Suleman K Rearrangements of the RAF kinase pathway in prostate cancer, gastric cancer and melanoma Nat Med 2010 16 7 793 798 20526349 10.1038/nm.2166 PMC2903732

[B6] Pflueger D Terry S Sboner A Habegger L Esgueva R Lin PC Svensson MA Kitabayashi N Moss BJ MacDonald TY Discovery of non-ETS gene fusions in human prostate cancer using next-generation RNA sequencing Genome Res 2011 21 1 56 67 21036922 10.1101/gr.110684.110 PMC3012926

[B7] Soda M Choi YL Enomoto M Takada S Yamashita Y Ishikawa S Fujiwara S Watanabe H Kurashina K Hatanaka H Identification of the transforming EML4-ALK fusion gene in non-small-cell lung cancer Nature 2007 448 7153 561 566 17625570 10.1038/nature05945

[B8] Bass AJ Lawrence MS Brace LE Ramos AH Drier Y Cibulskis K Sougnez C Voet D Saksena G Sivachenko A Genomic sequencing of colorectal adenocarcinomas identifies a recurrent VTI1A-TCF7L2 fusion Nat Genet 2011 43 10 964 968 21892161 10.1038/ng.936 PMC3802528

[B9] Salzman J Marinelli RJ Wang PL Green AE Nielsen JS Nelson BH Drescher CW Brown PO ESRRA-C11orf20 is a recurrent gene fusion in serous ovarian carcinoma PLoS Biol 2011 9 9 e1001156 21949640 10.1371/journal.pbio.1001156 PMC3176749

[B10] Morozova O Marra MA From cytogenetics to next-generation sequencing technologies: advances in the detection of genome rearrangements in tumors Biochem Cell Biol 2008 86 2 81 91 18443621 10.1139/O08-003

[B11] Metzker ML Sequencing technologies - the next generation Nat Rev Genet 2010 11 1 31 46 19997069 10.1038/nrg2626

[B12] Campbell PJ Stephens PJ Pleasance ED O'Meara S Li H Santarius T Stebbings LA Leroy C Edkins S Hardy C Identification of somatically acquired rearrangements in cancer using genome-wide massively parallel paired-end sequencing Nat Genet 2008 40 6 722 729 18438408 10.1038/ng.128 PMC2705838

[B13] Hampton OA Den Hollander P Miller CA Delgado DA Li J Coarfa C Harris RA Richards S Scherer SE Muzny DM A sequence-level map of chromosomal breakpoints in the MCF-7 breast cancer cell line yields insights into the evolution of a cancer genome Genome Res 2009 19 2 167 177 19056696 10.1101/gr.080259.108 PMC2652200

[B14] Forbes SA Bindal N Bamford S Cole C Kok CY Beare D Jia M Shepherd R Leung K Menzies A COSMIC: mining complete cancer genomes in the Catalogue of Somatic Mutations in Cancer Nucleic Acids Res 2011 39 Database issue D945 D950 20952405 10.1093/nar/gkq929 PMC3013785

[B15] Huret JL Dessen P Bernheim A Atlas of genetics and cytogenetics in oncology and haematology, year 2003 Nucleic Acids Res 2003 31 1 272 274 12520000 10.1093/nar/gkg126 PMC165573

[B16] Mitelman F Johansson B Mertens F Fusion genes and rearranged genes as a linear function of chromosome aberrations in cancer Nat Genet 2004 36 4 331 334 15054488 10.1038/ng1335

[B17] Zhang J Feuk L Duggan GE Khaja R Scherer SW Development of bioinformatics resources for display and analysis of copy number and other structural variants in the human genome Cytogenet Genome Res 2006 115 3–4 205 214 17124402 10.1159/000095916

[B18] Brazma A Hingamp P Quackenbush J Sherlock G Spellman P Stoeckert C Aach J Ansorge W Ball CA Causton HC Minimum information about a microarray experiment (MIAME)-toward standards for microarray data Nat Genet 2001 29 4 365 371 11726920 10.1038/ng1201-365

[B19] Hermjakob H Montecchi-Palazzi L Bader G Wojcik J Salwinski L Ceol A Moore S Orchard S Sarkans U von Mering C The HUPO PSI's molecular interaction format–a community standard for the representation of protein interaction data Nat Biotechnol 2004 22 2 177 183 14755292 10.1038/nbt926

[B20] Hucka M Finney A Sauro HM Bolouri H Doyle JC Kitano H Arkin AP Bornstein BJ Bray D Cornish-Bowden A The systems biology markup language (SBML): a medium for representation and exchange of biochemical network models Bioinformatics 2003 19 4 524 531 12611808 10.1093/bioinformatics/btg015

[B21] Burgoon LD The need for standards, not guidelines, in biological data reporting and sharing Nat Biotech 2006 24 11 1369 1373 10.1038/nbt1106-1369 17093486

[B22] Asmann YW Hossain A Necela BM Middha S Kalari KR Sun Z Chai HS Williamson DW Radisky D Schroth GP A novel bioinformatics pipeline for identification and characterization of fusion transcripts in breast cancer and normal cell lines Nucleic Acids Res 2011 39 15 e100 21622959 10.1093/nar/gkr362 PMC3159479

[B23] Edgren H Murumagi A Kangaspeska S Nicorici D Hongisto V Kleivi K Rye IH Nyberg S Wolf M Borresen-Dale AL Identification of fusion genes in breast cancer by paired-end RNA-sequencing Genome Biol 2011 12 1 R6 21247443 10.1186/gb-2011-12-1-r6 PMC3091304

[B24] Ge HY Liu KJ Juan T Fang F Newman M Hoeck W FusionMap: detecting fusion genes from next-generation sequencing data at base-pair resolution Bioinformatics 2011 27 14 1922 1928 21593131 10.1093/bioinformatics/btr310

[B25] Kinsella M Harismendy O Nakano M Frazer KA Bafna V Sensitive gene fusion detection using ambiguously mapping RNA-Seq read pairs Bioinformatics 2011 27 8 1068 1075 21330288 10.1093/bioinformatics/btr085 PMC3072550

[B26] Maher CA Kumar-Sinha C Cao X Kalyana-Sundaram S Han B Jing X Sam L Barrette T Palanisamy N Chinnaiyan AM Transcriptome sequencing to detect gene fusions in cancer Nature 2009 458 7234 97 101 19136943 10.1038/nature07638 PMC2725402

[B27] McPherson A Hormozdiari F Zayed A Giuliany R Ha G Sun MG Griffith M Heravi Moussavi A Senz J Melnyk N deFuse: an algorithm for gene fusion discovery in tumor RNA-Seq data PLoS Comput Biol 2011 7 5 e1001138 21625565 10.1371/journal.pcbi.1001138 PMC3098195

[B28] Sboner A Habegger L Pflueger D Terry S Chen DZ Rozowsky JS Tewari AK Kitabayashi N Moss BJ Chee MS FusionSeq: a modular framework for finding gene fusions by analyzing paired-end RNA-sequencing data Genome Biol 2010 11 10 R104 20964841 10.1186/gb-2010-11-10-r104 PMC3218660

[B29] Scherer SW Lee C Birney E Altshuler DM Eichler EE Carter NP Hurles ME Feuk L Challenges and standards in integrating surveys of structural variation Nat Genet 2007 39 7 Suppl S7 S15 17597783 10.1038/ng2093 PMC2698291

[B30] Kalas M Puntervoll P Joseph A Bartaseviciute E Topfer A Venkataraman P Pettifer S Bryne JC Ison J Blanchet C BioXSD: the common data-exchange format for everyday bioinformatics web services Bioinformatics 2010 26 18 i540 i546 20823319 10.1093/bioinformatics/btq391 PMC2935419

[B31] Beisvag V Kauffmann A Malone J Foy C Salit M Schimmel H Bongcam-Rudloff E Landegren U Parkinson H Huber W Contributions of the EMERALD project to assessing and improving microarray data quality Biotechniques 2011 50 1 27 31 21231919 10.2144/000113591

[B32] Field D Garrity G Gray T Morrison N Selengut J Sterk P Tatusova T Thomson N Allen MJ Angiuoli SV The minimum information about a genome sequence (MIGS) specification Nat Biotechnol 2008 26 5 541 547 18464787 10.1038/nbt1360 PMC2409278

[B33] Kottmann R Gray T Murphy S Kagan L Kravitz S Lombardot T Field D Glockner FO A standard MIGS/MIMS compliant XML Schema: toward the development of the Genomic Contextual Data Markup Language (GCDML) OMICS 2008 12 2 115 121 18479204 10.1089/omi.2008.0A10

[B34] Leinonen R Akhtar R Birney E Bower L Cerdeno-Tarraga A Cheng Y Cleland I Faruque N Goodgame N Gibson R The European nucleotide archive Nucleic Acids Res 2011 39 Database issue D28 D31 20972220 10.1093/nar/gkq967 PMC3013801

[B35] Shumway M Cochrane G Sugawara H Archiving next generation sequencing data Nucleic Acids Res 2010 38 Database issue D870 D871 19965774 10.1093/nar/gkp1078 PMC2808927

[B36] Yilmaz P Kottmann R Field D Knight R Cole JR Amaral-Zettler L Gilbert JA Karsch-Mizrachi I Johnston A Cochrane G Minimum information about a marker gene sequence (MIMARKS) and minimum information about any (x) sequence (MIxS) specifications Nat Biotechnol 2011 29 5 415 420 21552244 10.1038/nbt.1823 PMC3367316

[B37] Reese MG Moore B Batchelor C Salas F Cunningham F Marth GT Stein L Flicek P Yandell M Eilbeck K A standard variation file format for human genome sequences Genome Biol 2010 11 8 R88 20796305 10.1186/gb-2010-11-8-r88 PMC2945790

[B38] Sayers EW Barrett T Benson DA Bolton E Bryant SH Canese K Chetvernin V Church DM Dicuccio M Federhen S Database resources of the National Center for Biotechnology Information Nucleic Acids Res 2012 40 Database issue D13 D25 22140104 10.1093/nar/gkr1184 PMC3245031

[B39] Kim P Yoon S Kim N Lee S Ko M Lee H Kang H Kim J ChimerDB 2.0--a knowledgebase for fusion genes updated Nucleic Acids Res 2010 38 Database issue D81 D85 19906715 10.1093/nar/gkp982 PMC2808913

[B40] Nakaya J Kimura M Hiroi K Ido K Yang W Tanaka H Genomic Sequence Variation Markup Language (GSVML) Int J Med Inform 2010 79 2 130 142 19969503 10.1016/j.ijmedinf.2009.11.003

